# Commercialisation and commodification of breastfeeding: video diaries by first-time mothers

**DOI:** 10.1186/s13006-020-00264-1

**Published:** 2020-05-01

**Authors:** Alison M. Taylor, Jo Alexander, Edwin van Teijlingen, Kath M. Ryan

**Affiliations:** 1 Centre for Midwifery, Maternal & Perinatal Health, Faculty of Health and Social Sciences, Bournemouth University, Royal London House, Christchurch Road, Bournemouth, Dorset, BH1 3LT UK; 2 Centre for Midwifery, Maternal & Perinatal Health, Faculty of Health & Social Sciences, Bournemouth University, Bournemouth, UK; 3 School of Pharmacy, University of Reading, Whiteknights Campus, Po Box 226, Reading, UK

**Keywords:** Qualitative; commercialisation of breastfeeding, Experiences, United Kingdom, Audio, Visual

## Abstract

**Background:**

Many of aspects of our lives became increasingly commercialised in post-modern society. Although breastfeeding is perhaps a late comer to this process in recent years, it too has seen significant commercialisation facilitated by social media and our obsession with celebrity culture. This paper explores how the commercialisation and commodification of breastfeeding impacts mothers’ experiences of breastfeeding.

**Methods:**

In a qualitative study, five mothers in the United Kingdom recorded their real-time breastfeeding experiences in video diaries. Using a multi-modal method of analysis, incorporating both visual and audio data, a thematic approach was applied.

**Findings:**

Women preparing for breastfeeding are exposed to increasing commercialisation. When things do not go to plan, women are even more exposed to commercial solutions. The impact of online marketing strategies fuelled their need for paraphernalia so that their dependence on such items became important aspects of their parenting and breastfeeding experiences.

**Conclusions:**

The audio-visual data demonstrated the extent to which “essential” paraphernalia was used, offering new insights into how advertising influenced mothers’ need for specialist equipment and services. Observing mothers in their video diaries, provided valuable insights into their parenting styles and how this affected their breastfeeding experience.

## Background

“Pregnancy today is highly visible, intensely surveilled, marketed as a consumer identity, and feverishly stalked in its celebrity manifestations” [1]. These images, from Tiidenberg et al. spill over to breastfeeding where celebrities have used visual statements to share their thoughts about feeding their babies. A recent social media example was a photo shoot for *Girls Girls Girls* magazine of Rachel McAdams, celebrity actress wearing chic Versace clothes, Bulgari jewellery and using a double breast pump [2]. She was reported to have said “if you’re a stylish mum to a 6-month-old baby, you have to grab your breast pump in between takes” [2]. With hashtags including “#normalise breastfeeding” and nearly 100,000 likes (December 2018) and over three thousands comments on the Instagram post about breastfeeding and breast pumps [2], this photo received much attention.

There is a plethora of research evidence about how the known health benefits of breastfeeding can create a moral obligation for women to breastfeed to be considered by society as ‘good mothers’ [3–6] and expressed breastmilk (EBM) can help them fulfil this ‘obligation’ [6–8]. One study discussed the use of EBM as a commercialised commodity so that “women became users (and their babies consumers) of their own product” facilitating father-infant bonding to occur and a sense of autonomy for women to return to their social and public lives [7]. McInnes et al. found that UK websites portrayed EBM with conflicting messages since commercial websites marketed pumping as a means of solving parents’ apprehensions, including restrictions on breastfeeding, and non-commercial websites focused on broader aspects of expressing including hand expression [9]. Commercial messages portrayed in breast pump advertisements romanticised the natural process of pumping with a close relationship between mother and baby despite the baby not feeding from the breast [10]. Thus, whilst there is evidence of the commercialisation and commodification of breastmilk as a product from varying perspectives there is less evidence exploring mothers’ perspectives of other commercial influences on breastfeeding.

This study explores the commercialisation and commodification of breastfeeding that influence mothers’ daily experiences of breastfeeding within their own social environment to enhance understandings of the socio-cultural context of breastfeeding in the UK.

## Methods

In qualitative research, the researcher often explores perspectives of participants [11]. Ethnography involves continuous contact with a specific cultural group over a period of time, usually involving observation of their actions and interactions within the context of daily living and focusing on how they make sense of their world including what they perceive about their reality [12]. Ethnographic studies to explore women’s experiences of breastfeeding have been undertaken on postnatal wards [13, 14]. These studies provided insights into how women started their breastfeeding journeys and how the medicalised environment in hospital impacted on the care provided and the resultant effects on their breastfeeding experiences. Ethnographic research exploring women’s experiences of breastfeeding in the community observing their interactions with health professionals suggests feeding choices are influenced by social, practical and emotional factors [5]. However, the observations appeared to have been recorded at one episode of interaction between a woman and a midwife, the research did not provide a continuing exploration of each woman’s breastfeeding experience on a daily basis over a period of time in the home environment. Other ethnographic studies have explored women’s breastfeeding experiences in public settings: child health clinics [15] and a mother/baby support group [16]. Tomori undertook an ethnographic study exploring breastfeeding in the home but the study was limited to participant observation in the day. It did include data concerning night-time feeding gathered from the conversations observed [17].

Video ethnography has been used in the maternity services to capture footage of care during and after caesarean section. This enabled multiple viewing for in-depth analysis [18] but presented challenges such as the “inability of the researcher and camcorder to blend into the environment” and remain unnoticed [19]. Pink and Mackley [20] explained how ethnographic research using the “video tour” method provided insights into “how the sensory aesthetic of home is experienced, produced and maintained” in everyday domestic life. Bates used video diaries in an ethnographic study exploring the body, health and illness in daily life with people with long-term physical or mental health conditions [21]. Although a physical object, the camera acted as the “participant observer” with “eyes and ears” for the participants to talk to [21]. In our study, video diaries using an ethnographic approach offered the opportunity for mothers to record their holistic expressions through word and action, their embodied ways of knowing and experiencing breastfeeding within their own socio-cultural environment and the private space of their own home. This approach was considered practical, ethical and sensitive [22, 23] and not intrusive to participants.

A pilot study involved one breastfeeding mother and baby, which included assessing whether the sampling and recruitment strategy were useful, and the camcorder along with some simple guidance was an effective research tool [24]. The pilot resulted in more than 11 h of rich data which were therefore included in the main study [25]. Published studies involving video diaries recruiting between three [26] and 20 participants [27] have been shown to produce abundant data and the pilot study confirmed both the breadth and depth of data that could be collected. English-speaking women living in the South of England between 28 and 30 weeks pregnant with their first child were invited by midwives to participate. The primary researcher visited respondents at home at 32–35 weeks gestation to gain informed consent and offer two-weeks’ practice with the camcorder. This first meeting was intended to build “trust and rapport” so that potential participants felt comfortable sharing their experiences [28]. Whilst it is not possible to identify how many women were invited by midwives to participate, seven requested a home visit to discuss the study before consenting to take part. The camcorder was returned to the mother after the birth when she and the full-term baby were confirmed healthy and she was breastfeeding. Three of the seven women who had consented no longer fitted the inclusion criteria [25]; one was fully formula feeding prior to discharge, one had a preterm birth, and one mother was unwell.

Five breastfeeding mothers, including the pilot participant, recorded daily videos on any aspects of breastfeeding they considered worth mentioning for the first few weeks following birth until their preferred method of feeding was established. Along with the camcorder, a one-page guide for the video diaries was provided to stimulate conversation and recordings [29]. Participants were encouraged to record at least one daily monologue and to talk for as long or as short as they liked, including on breastfeeding or demonstrating the equipment they were using. All participants were shown how to view and edit their videos prior to providing the data which were collected every 1–2 weeks when written consent was given to release the data for analysis and publication.

### Analysis

The ethnographic stance meant that we did not just focus on the audio content of the video but used both visual and audio ethnographic approaches to analyse verbal and non-verbal behaviours, language, values, and how other people influenced mothers’ perspectives. Interpreting cultural behaviours also involved analysing what mothers included and what was left out of the frame, including whether they recorded themselves breastfeeding or expressing breastmilk, or avoided these aspects altogether. This ethnographic stance also involved analysis of the mother’s breastfeeding environment including features that influenced and affected their breastfeeding experiences including furniture, clothes, equipment and people present.

The principal researcher started the five phases of the thematic analysis with a “familiarisation phase”, observing each participant’s video clips sequentially, with little disruption between diary entries, to help her become immersed in all aspects of the mother’s journey [30]. On second viewing handwritten notes were made with essential content including the woman’s environment, verbal and non-verbal behaviour and discussion content. This content was typed up as outline logs [31] and helped create initial impressions pertinent to the research question and the identification of some connecting and contrasting patterns [32]. NVivo software was used [33] to avoid the potential loss of data through the complicated and erroneous job of simultaneous transcribing of audio and visual data [34]. The next phase involved “complete coding” of the whole data set [30] by viewing and clipping the video files numerous times and in numerous ways to digitally link them to codes. Following this stage of the analysis, when no new codes were discerned and there were a variety of examples within each code, it was decided that there was no need to collect further data. The resulting 170 codes were then combined to form patterns of meaning [35]. Using iterative cycles of analysis, clusters of codes were gathered into categories, subthemes and themes [30].

A reflexive approach was important throughout the whole research process [36] which included data collection as the research team were aware that although they were not present when mothers recorded their daily diaries, they were implicit in the research study as usually the mothers addressed them directly [37]. Reflexivity also meant that when coding, analysing and interpreting the data the first author was more mindful that her interpretations might have been influenced or biased by her own personal and professional background as a midwife and mother [12]. Thus all members of the research team aimed for ‘empathic neutrality’ by being open, sensitive and respectful in their approach to the analysis [38]. The credibility of the analysis was also enhanced by the different interprofessional perspectives of the co-authors who sampled the video clips to check that the first author’s initial interpretations were accurately captured [32]. Additionally, concerted efforts were made to remain close to the data by representing mothers’ experiences using their own words.

This paper focuses on the commercialisation and commodification of breastfeeding from the perspective of individual mothers arising from the theme ‘essential paraphernalia’. The findings are illustrated with quotes from the mothers. Pseudonyms have been applied throughout followed by the age of the baby in weeks.

## Results

All participating breastfeeding mothers were British Europeans aged between 20 and 29 years and co-habiting or married. Using the Standard Occupational classification [39] before the birth, the women were classified in groups 2–10 with group 10 added for unemployed/student [25].

It appeared important to the mothers to record that breastfeeding continued around the clock and what their experience felt like. By recording issues related to breastfeeding as it was happening, the mothers provided a ‘real-time’ perspective of breastfeeding that has not been captured before. The spontaneity of recording provided evidence of the impulsive nature with which four of the participants turned on the camcorder without there being a sense of planning or forethought. The diversity in timings, quantity and frequency of recordings demonstrated the commitment of mothers to the research. The purposive sample of five participants recorded 294 video entries lasting 43 h and 51 min, provided an abundance of rich data (see Table 1). Consequently, further participants were not recruited.

**Table 1:** Video diary recording details for each mother – Original source [25]

Participant Pseudonym	Average length of a recording	Number of days/weeks recorded over	Max number of recordings in any one day	Shortest recording	Longest recording	Total time recording	Total number of recordings
Rosie	18 min	63 days 9 weeks	3	9 s	43 min 20 s	14 h 59 min 35 s	51
Sam	4 min 48 s	48 days 6^+ 5^ weeks	2	1 min 49 s	20 min 38 s	1 h 26 min 32 s	18
Sarah	8 min 22 s	87 days 12^+ 3^ weeks	2	1 min 10 s	32 min 48 s	4 h 19 min 36 s	33
Tracey	12 min 33 s	130 days 18^+ 4^ weeks	6	27 s	57 min 42 s	11 h 42 min 52 s	71
Vicky	8 min 9 s	123 days 17^+ 4^ weeks	8	17 s	1 h 12 min 45 s	11 h 23 min 3 s	121

### Thinking ahead

The video diaries allowed mothers to work out their challenges by discussing, reflecting and sharing thoughts and feelings about breastfeeding. All felt the pressure that they should breastfeed to be a good mother because they had been informed of the health benefits of breastfeeding for their baby but no-one had warned them of the potential challenges of constantly feeding 24 h a day. Some mothers, thinking ahead during pregnancy about breastfeeding, bought commodities that might make it easier. These commodities did not always meet expectations, for example, Rosie acquired a special cot that attached to her bed for easy access for breastfeeding. She presented it in one of her pre-birth practice videos, but subsequently said:

*[Lying on bed cuddling Lily on chest] … in the night time, Lily won’t sleep in this [pointing] fabulous little co-sleeping cot that is attached to the bed, she wants to be right next to me, so she sleeps in the crook of my arm which I know is technically highly illegal and everything but it seems to be the only way we can get any sleep* … (Rosie1)

Social media banning breastfeeding pictures affected participants, so that they felt reserved about breastfeeding in front of strangers, believing it was socially unacceptable.

*I bought this before Sophie was born … it’s a breastfeeding cover [showing the camera] … and you are supposed to put it over your shoulder … to sort of hide yourself while you are feeding, which is all very well, but I find it REALLY fiddly, it almost draws MORE attention because it’s like a different colour to your clothes, and also it goes over Sophie’s head … [demonstrating over her own head] … and I feel like it restricts her movement and makes her feel probably a bit claustrophobic [screwing face up]* (Sarah6)

### Marketing influences

During pregnancy, the mothers felt lured into buying a whole array of paraphernalia from one specific manufacturer believing its slogan that this equipment was as near to breastfeeding as possible. For Sam, this included bottles, teats, steriliser, bottle warmer, monitor and breast pump. She was devastated when the steriliser and the breast pump failed within 3 weeks and the teats did not live up to her expectations. Experiencing engorgement when her breast pump failed because she had been pumping and breastfeeding caused Sam to feel angry and let down.

*We also had our breast pump break, which I am REALLY annoyed about because it is BRAND new. It just doesn’t pump [shaking head] there’s no suction [shaking head] so obviously I’ve got to take that back, but when things like that happen, I find it really hard because it means I can’t express, which means my breasts hurt* (Sam3)

### Searching online for solutions

All mothers used the internet to ‘look up’ information when problems arose, when they had unanswered questions or needed to validate or reassure themselves.

*Again, my nipple is severely sore … not quite sure what to do next. I have been looking online for things to do. I have seen something called nipple soothers. So, I’m going to look in the shops for those. See if I can repair it rather than having to give up feeding* (Sam2)

Using the internet meant that they could look up information instantaneously as a self-help method of answering questions before seeking advice from health professionals. Searching the internet inevitably meant that mothers were daunted by an overwhelming number of diverse websites including those sponsored by the commercial companies producing bottles and teats. Tracey, who spent a great deal of time searching for information about expressing, recognised this commodification and was disappointed. She was not looking for the best buy but interested in learning about the process. Maybe she had the insight to realise the potential bias of the commercial information or maybe the information did not answer her questions so that she became frustrated.

*I was looking on the internet the other day … because I wanted to know about expressing … and there really wasn’t anything out there except for the manufacturers’* [websites for pumps] (Tracey6)

Looking for answers to their breastfeeding challenges brought the mothers into contact with the commercialisation of breastfeeding. Together they bought an array of equipment from nursing clothes and bras, breast pads, breastfeeding covers and pillows, baby slings, novelty cradles and rockers, nipple soothers and shields, breast shells, breast pumps, bottles and teats, all claiming to make breastfeeding or parenting easier. The mothers presented these specialised commodities because they considered them either indispensable for breastfeeding or did not meet their needs or expectations.

The yearning to have independent time and space away from their baby was experienced by all but Rosie. The others spent time working out how to share feeding, how to express and what bottles and teats to use and searching online for the perfect solution. Various pumps were demonstrated with one mother giving a step-by-step demonstration of how milk was expressed from her breast.

Three mothers preferred specific brands that they promoted as the best, suggesting marketing strategies did not just attract the mothers to one product, but to a whole array of products that they felt they needed.

*We have found the company [brand name] to be fabulous, I have their breast shields, electric breast pump and the [manufacture’s slogan] teats for the bottles, which Peter is using to feed my breastmilk about once a week, so it’s slightly more flexible than him [baby] only taking the breast* (Vicky8)

Sam started expressing breastmilk on day six after the birth when she left baby Zac with her own mother to attend a social party on day eight. At 13 days, she started to plan going back to college and returned for the first time on day 22. She had no support from health professionals with this planning and, despite limited income, bought three pumps and with trial and error found her own method to achieve her goals.

*The other thing we bought … was my electric breast pump [holding up in front of camera and switching it on], it sounds a bit scary [laughing] it is scary. When I first used it, I was really nervous thinking that it would suck too hard or something would happen, but amaaaazing, best thing we ever bought, better than the hand pump, just sucks in a different way, I’ve actually got some ridiculous amounts of milk now* (Sam7)

Perhaps not recognising the conflicts of interest on commercial websites, some mothers reported their findings with conviction, believing that what they had read was from a trustworthy source.

*… you can get this thing called nipple confusion … all this stuff I’m learning, it’s brilliant … I’ve been looking at the website [brand name] and they’ve made a new breast teat which means they [babies] have to do the same sort of feeding as when you are breastfeeding* (Vicky4)

Nipple/teat confusion was a concern for more than one mother and so finding the right teat was a particularly important aspect of mothers’ breastfeeding experience. Despite craving space from their baby, they were keen that breastfeeding was not disrupted by using a bottle of breastmilk and that the bottle-feeding system did not leak or waste breastmilk. Sam having bought some ‘*new little gadgets*’ expressed her eagerness to show the specialised ‘breastfeeding teats’ pointing out their unique ‘selling features’.

*...we actually got a specialised breastfeeding teat and bottle which has a main teat and then a little teat inside [demonstrating] and that enables the baby to make their own shape inside of the teat, which would be the same way that they form your breast. We have found this is amaaaazing...he can’t spill any of my valuable breastmilk* (Sam3)

### Paraphernalia which became essential

Some mothers became completely dependent on their breastfeeding paraphernalia including breast shields and cream, suggesting that they would not have been able to continue breastfeeding without them, even when their infant feeding method was established.

*I’m still using the lanolin cream … for my nipples, I don’t actually need it now ...I think it’s habit and I think where I got so sore before, I’m so afraid of getting sore again I just use it all the time and it’s very expensive, it’s £10 a tube* (Sarah9)

Mothers talked about ‘putting their baby down’, and took pride in showing cribs, cots, vibrating and bouncy baby chairs and baby gyms. The mothers used equipment to gain some distance and space from their baby, and time to engage with other activities.

*I can have a shower because he’s in his chair, cooing at himself... It’s the best twenty quid I have ever spent in my life because he gets to be a little bit happier* (Tracy6)

Making time and space away from her baby was not Rosie’s priority, and although her partner had mentioned fleetingly about giving Lily a bottle of EBM, this was considered as *‘second best’* (Rosie7). For her, breastfeeding was a continuum of pregnancy and she rarely videoed without her baby being attached, either breastfeeding, in her arms or in the sling. Rosie recommended a sling as part of every mother’s essential equipment as Lily stayed settled for longer periods between feeds in the sling and Rosie’s hands were free for other jobs. Talking about this on several occasions, and then switching the camera on to purposefully demonstrate its use, she appeared to take great delight in demonstrating how her sling worked.

*… how fantastic is this sling? … Surely that ought to be part of what the midwife and the health visitors show you or recommend?* (Rosie10)

Bras (good breast support), breast pads (deal with leaking breastmilk) and clothes (easy access for breastfeeding) were important paraphernalia. Going shopping was more difficult once the baby was born and online shopping was confusing with a vast array of bras available. Representations of breastfeeding on commercial websites were considered ironic as these did not reflect the mothers’ reality of breastfeeding.

*… feel it would be a really good idea to buy some breastfeeding bras … [shaking head] it’s like so baffling there’s so many different kinds … it’s hard to know like what to look for in it … spent quite a long time trawling about on different websites laughing at these incredible models who don’t [laughing] remotely look like nursing mothers and in the end I haven’t bought anything at all yet.* (Rosie1)

Prolific leaking was a major problem for some. They promoted their favourite brands of muslin cloths, washable and disposable pads and breast shells as if they were appearing on a television advertisement. Perhaps, since they had given consent for us to share our findings including video clips in publications, educational resources and broadcasts, on the Internet and at conferences, the participants thought that part of their role was to provide recommendations about specific products.

*… they are amazing … the actual breast pad themselves [holding up to camera] you probably can’t even see, but it is like silk, on the inside, really soft, and I found all the other breast pads leak, if they get too wet, which I don’t like [shaking head and frowning] … I highly recommend them … cost a bit more, but it’s worth the money* (Sarah3)

Elsewhere in a video clip Sarah refers to the concept of value for money:

*… [brand name] are making a fortune out of me, I bought these today [reading from box of breast shells], they catch your milk ...they were £11, but I think they are worth every penny if they catch the precious milk that I can get out of me rather than giving her formula rubbish …* (Sarah3)

The online market for the product that would provide ultimate relief from leaking was confusing for Rosie who wanted an ethical method to prevent everyone seeing her *‘tide marks left behind from leaking breastmilk’*. With such a vast array of products marketed online, including disposable pads, reusable pads made with bamboo, silicone, microfibre polyurethane laminate and organic cotton, pads with fashion designs and collection cups, pumps and shells, it is not surprising the women bought several commodities to manage this problem. Maternity bras and clothes that facilitated easy access and ‘latching’ were important to mothers. Rosie invested in two tops for easy access, but despite feeling they helped, she was still seen grappling with layers of clothes in her video diaries.

*… nursing tops have arrived finally … there’s a cross bit, and a bit that comes up … it’s a bit less drafty and a bit more modest as well, it’s a success (*Rosie5)

## Discussion

Unexpected breastfeeding challenges made mothers vulnerable to commercial influences as they sought instant solutions on the internet to make sense of their unnerving experience. Being born in the 1980s meant that the mothers grew up with instant internet access on their phone, tablet or computer. Studies have revealed that women take advantage of the immediate accessibility of online health information during pregnancy [40] particularly when there is a lack of information provided by health services [41, 42]. In one study, during a 12-week period, 97% of 613 pregnant women from 24 countries were found to have used search engines to access information about pregnancy, find support groups and shop online [42]. The internet is an unregulated source of information and there is no guarantee that people have the skills required to evaluate whether what they are reading is authentic, trustworthy, correct, valid or appropriate: this lack of necessary skills can result in erroneous decisions and poor outcomes [42, 43].

The mothers in this study found information from a diverse range of websites including commercial ones. Only one mother (Tracey) suggested she was aware of the potential bias of the information she was viewing. Shaikh and Scott found that consumers in the United States most frequently used websites sponsored by commercial enterprises with a propensity to include advertisements on educational material, breastfeeding supplies and other items [44]. Lima-Pereira found that almost 70% of participants trusted the information that they viewed even though they were more likely to use commercially sponsored websites than non-commercial ones [41]. While one study deemed most of the content on the websites was correct and conformed to the International Code of Marketing of Breast Milk Substitutes [41], another found that very few had all information accurately presented [44]. In addition, the increasing sophistication of the digital marketing industry means that mothers who used search engines to look at commodities online found adverts for the same commodities popping up repeatedly on social media and other forums with the aim of tempting them to buy the product [45]. Currently, there is little published evidence about the influence marketing and commercial websites have on mothers’ infant feeding choices and practice.

This study demonstrated through visual and audio data the importance that mothers placed on expressing breastmilk using a pump and the early introduction of a bottle. Some mothers expected opposition to their ideas from the healthcare team, and so searched the internet to gain some understanding and to find the right equipment to pursue their goal. Thomson and Dykes reported health professionals’ reluctance to share information about bottles, teats and nipple shields in an evaluation of the implementation of the Unicef UK Baby Friendly Initiative (BFI) standards in the community, despite mothers maintaining that these items were essential for breastfeeding [46]. Hence these researchers warned that when healthcare workers limit information, it can lead to misunderstanding and the potential misuse of equipment resulting in complete cessation of breastfeeding.

Introducing a bottle of EBM was seen as a way of managing the problems that occurred in the early days, such as painful nipples, mastitis and a perceived inadequate milk supply, but mainly to fulfil the craving for some freedom and independence [8, 47, 48]. This need for freedom and independence partly revolved around the necessity to work out the logistics in relation to returning to work or college [49] and partly an altruistic drive to involve the father who also wanted to experience feeding his baby [50, 51]. These findings support the growing discourse in the literature highlighting the dissonance between the ‘reciprocal, instinctive, embodied’ nature of breastfeeding and the mothers’ imperative to control and manage the process to create some sort of ‘routine’ and ‘freedom’ in daily life [7, 52]. This latter approach is thought to have been fuelled by the medical model of infant feeding which started at the turn of the twentieth century promoting strict schedules, feeding measurements and regular inspection of infant growth and development, the influence of which is still strong today [24, 53, 54]. This medicalised approach, apart from promoting formula milk, has objectified breastfeeding through focusing more on the benefits of breastmilk as a product than the process of breastfeeding [55, 56]. Breast pumps have been identified as commodities endorsing that approach [57].

The market for commodities is dependent on new ‘needs’ being identified [58]. Van Esterik warns that commercial companies target new mothers through advertising breastfeeding essentials. The result is the purchasing of many unnecessary consumer goods with the anticipation of something going wrong [59]. This medical model of infant feeding has provoked a technological approach to breastfeeding which has created the ‘need’ for commercial companies to produce the ‘perfect’ double pump, for example, which enables a mother to do virtually anything else but breastfeed her baby while expressing, such as in the photo shoot of Rachel McAdams [2]. In addition, the internet has provided mothers instant access to online shopping for commodities with next day delivery if required. It is not surprising then that mothers in this study were enticed into the commercial world of breastfeeding when they went on the internet or spoke to friends. With manufacturers' slogans shared in their video diaries about the equipment they bought, and demonstrations in front of the camcorder promoting their chosen products and brands, they appeared convinced that they had found the best product and that it was essential for them.

Douglas, in a theoretical discussion about women’s rite of passage as breastfeeding mothers, argued that they are currently caught between the ‘medicalisation of infant feeding’ and ‘yummy mummy’ syndrome [60]. The former results in women being hindered by biomedical surveillance by health professionals, triggering a mistrust of their own body’s ability to breastfeed. The ‘yummy mummy’ syndrome is part of post-modernity which encourages mothers to become empowered and take control of the physical ‘transfiguration’ of their bodies so that they become fit, healthy and ‘gorgeous’ with anything less considered a failure. Remarkably similar to Douglas’ icon of the postmodern ‘milkmother’ [60], Blum warned that the breastfeeding ‘Supermom’ syndrome in America was the product of medicalisation combined with commercialisation of breastfeeding. The outcome was that mothers expected to work and meet the standards of the ‘yummy mummy’ [61]. Arguably, the photoshoots of celebrities are iconic reminders of the ‘Supermom’ syndrome. The commodities on the internet promoting an ‘easier’ breastfeeding experience appealed to mothers in this study who required a balance between work, rest and play. This requirement included, for some, aspirations for ‘supermom’ status, multi-tasking, resuming social life and dressing up to go out, at the same time as trying to reduce the anxiety engendered by health practitioners’ medicalised approach to support.

Once expressing was underway, mothers needed to “train” their babies to take breastmilk from the bottle as well as from the breast [49]. This caused tensions between introducing a bottle before it was too late because of a fear that rejection later would stifle independence and freedom, and a fear of nipple/teat confusion [62]. The conflicting opinions about nipple/teat confusion have caused much debate in the literature [63] even though its avoidance is one of the main rationales underpinning Step 9 of UNICEF Baby Friendly Hospital Initiative (WHO 1998) which prohibits the use of teats or dummies for breastfed infants. Seemingly, commercial companies have used this apparent ‘need’ [58] to create an array of teats specially designed for the breastfeeding baby which according to Hilton [64] have been developed as a result of research using ultrasonography to view babies’ suckling at the breast. The conflicting opinions to which mothers were exposed, including vicarious experiences of friends and family, scientific evidence from health professionals or books, and commercial advertising on the internet caused much debate in the video diaries. When mothers were searching for the ‘perfect’ solution to prevent nipple/teat confusion, the commercial websites apparently reassured them that they had the answer.

Applying Kirkham’s [58] description of ‘commodification’ further, it became apparent from the video diaries that commercial companies have sought to use breastfeeding and some of its perceived challenges for their commercial gain. Leaving breast pumps and bottles aside, mothers presented a wide range of different commodities that they considered indispensable to their breastfeeding experience. This array of specialised equipment has also been highlighted in an American study interviewing 25 first-time mothers from privileged backgrounds who were thought to have a greater chance of continuing breastfeeding [65]. Indeed, likening it to a “project”, Avishai highlighted the expensive obsession women had for buying the latest gadget [65]. In contrast, this study did not involve parents on very high incomes and yet the trend for buying breastfeeding related paraphernalia was evident. Kirkham highlighted the exploits of commercial companies to lure, through advertising, new mothers who can least afford to buy their commodities because of the apparent ‘need’ for the new baby to fit into contemporary lifestyle choices [58].

Consequently, commercial companies through clever marketing suggest that everything can be fixed at a price, have monopolised the way contemporary mothers think of themselves, create new identities, and manage their breastfeeding experiences. Findings from this study demonstrated how commercialisation and commodification affected mothers’ experiences without them realising it, including exacerbating the potential issues that could undermine their confidence in breastfeeding.

Breastfeeding in public was one of the challenges that some mothers faced, as they were embarrassed and felt ‘out of place’ (Sam1). This challenge has resulted in commercial companies marketing specially designed bras and clothes like the top Rosie bought designed for the “conscious … with a wrapover front and practical inner top for easier nursing access” [66]. Of the five mothers, Rosie was the least concerned about breastfeeding in public but nevertheless bought one of these garments. In addition, breastfeeding “udder covers”, originating in America and similar to the one demonstrated to the camera by Sarah, have been designed to help mothers to “breastfeed baby and still socialize with family and friends confidently” - “all while wearing something chic and stylish” [67]. The phrase “udder covers” could be considered a form of black humour by some but emphasises the paradox faced by breastfeeding mothers. There is a moral imperative to breastfeed alongside the pressure to maintain a ‘yummy mummy’ appearance to conceal the ‘animalistic’ natural function of the maternal body. We would argue that advertising very cleverly reinforces the ‘modern’ discourse of moulding mothers’ desires to become ‘yummy mummy’ models while offering ‘solutions’ for unrealistic and uncomfortable aspirations which seriously undermine mothers’ confidence. Women are having unrealistic expectations and aspirations of themselves as new mothers, making them easy prey for advertisers. Additionally, advertised solutions add to their notions of failing as perfect mothers. Advertising has the potential to fuel guilt and failure when mothers are unable to conform to what they think are the standards set by society.

Leaking breastmilk is another breastfeeding ‘problem’ that companies have used to create a commercial ‘need’. Concurring with previous research, mothers in this study found leaking breastmilk annoying, embarrassing, awkward or uncomfortable [68, 69], especially when it was prolific. Whilst this study highlights the uncontrollable, neuro-hormonal, humorous and private nature of the let-down reflex with ‘spraying’ and ‘leaking’ of breastmilk witnessed on camcorder, it also captured the embarrassment mothers felt and their attempts to hide from the gaze of others. Mothers promoted different brands not just for comfort, but as Battersby suggested, to conceal leaking milk so that they were not embarrassed or judged as dirty or offensive [70].

Relentless breastfeeding and an unsettled baby were interlinked. This study illuminated the way mothers bought and used some commodities aimed at settling their baby, providing valuable insights into their differing parenting styles and their impact on their breastfeeding experience. Some mothers, for example, took pride in filming their babies asleep in rocking or vibrating chairs, which are promoted on the internet as “the ultimate nap space for baby … plenty of relaxing features for good-quality sleep” [71]. Exhausted mothers invested in these commodities to gain some ‘space’ from their baby. It could be argued that if the manufacturers’ ‘promise’ was fulfilled, their babies would have slept for longer and feeding cues might have been missed, potentially undermining the natural physiology of lactation which is dependent on responding to baby’s early feeding cues and frequent feeding. In contrast, ‘co-sleeping cots’ are advertised by commercial companies as encouraging ‘closeness, safety and convenience’ for easy night-time feeding because newborns are easily accessible but have ‘their own space’ for sleeping [72]. During pregnancy this appeared to Rosie to be a culturally acceptable way of keeping her baby close at night. However, as Tomori identified [17], the thought of moral judgement by others about co-sleeping was challenged when it became more intuitive and effective to bring her baby into bed with her for breastfeeding and sleeping. Commercial companies have also recognised that ‘baby wearing’ for the “busy mum” is becoming popular “for helping parents to bond with and soothe a new baby … and to enable breastfeeding on the go” [73]. Following an “attachment mothering approach” [74], Rosie mostly recorded herself either feeding or baby wearing at the same time as multitasking, and could not understand why slings were not recommended for all mothers. Evidence suggests that breastfed babies carried in slings are less likely to cry and more likely to gain weight [75]. Arguably, using a sling embraced the “supermom” syndrome, while keeping baby close enhanced the neuro-hormonal response and the maintenance of lactation. However, the use of slings is contentious; the Department of Health’s publication (2011) ‘Off to the Best Start Leaflet’ still showed a picture of the way Rosie used her sling initially despite no longer being recommended because of serious health risks [76]. This sling has since been withdrawn from the market and websites selling slings frequently display safety rules for baby wearing.

### Limitations

The findings of this study are based on five British European women living in the UK and, therefore, may not be generalizable. The sampling strategy limited recruitment to first-time mothers to avoid previous experience influencing their subsequent perceptions and experiences of breastfeeding [77]. This sampling strategy means that the findings are not necessarily transferable to breastfeeding mothers who have breastfed before.

Since the first author was working with the participants in the capacity of a researcher rather than as a practising midwife, it was important that participants understood that breastfeeding advice could not be provided to the study participants. This restriction reflected the purpose of the research which was to explore the type of support a mother would seek and how easy or difficult obtaining that support would be. On first viewing of the tapes and visiting the family at home, the first author looked for psychological stress caused by recording and/or editing the video diary [78]. If this had been apparent, the data collection would have stopped and referral to specialist support offered. Whilst there were a host of emotions expressed by the participants on camera, they did not appear to be a result of recording alone but rather the result of their experience. Indeed, as reported earlier, talking to the camcorder was felt by the mothers to be a positive cathartic release [25]. However, the first author fully understood that when visiting mothers, her duty of care needed to override her role as a researcher; in the name of safety such as an obstetric emergency, immediate and appropriate action would have been delivered [78, 79].

## Conclusion

Mothers searched the internet for breastfeeding information and equipment to assist them with their daily challenges. The audio-visual data demonstrated the extent to which the paraphernalia were used, offering new insights into how advertising lured mothers into thinking that they were dependent on specialist equipment. The audio-visual data provided valuable insights into the parenting styles that mothers adopted and how this affected their breastfeeding experience.

The data highlighted the frequent need mothers felt to use the internet if they did not get their queries answered by healthcare workers on how to manage unforeseen challenges associated with breastfeeding. Searching the internet exposed mothers to commodities promoting the ‘yummy mummy’, with its emphasis on looking stylish and hiding all signs of breastfeeding. This undermined the mothers’ confidence and generated unrealistic expectations with the potential to fuel guilt and failure when they felt they were not conforming to their desired socio-cultural standards. When exhausted, mothers invested in commodities that had the potential to undermine breastfeeding through promoting separation from their baby and ‘buy in’ to the ‘supermom’ culture, causing further exhaustion and feelings of failure.

## Data Availability

The data that support the findings of this study are available on request from the corresponding author [AT]. The data are not publicly available due to them containing information that could compromise research participant privacy.
